# New Freeze-Dried Andean Blueberry Juice Powders for Potential Application as Functional Food Ingredients: Effect of Maltodextrin on Bioactive and Morphological Features

**DOI:** 10.3390/molecules25235635

**Published:** 2020-11-30

**Authors:** Mauren Estupiñan-Amaya, Carlos Alberto Fuenmayor, Alex López-Córdoba

**Affiliations:** 1 Facultad Seccional Duitama, Escuela de Administración de Empresas Agropecuarias, Universidad Pedagógica y Tecnológica de Colombia, Carrera 18 con Calle 22 Duitama, Boyaca 150461, Colombia; maurenest01@gmail.com; 2 Instituto de Ciencia y Tecnología de Alimentos (ICTA), Universidad Nacional de Colombia, Av. Carrera 30 # 45-03, Bogota 111321, Colombia; cafuenmayorb@unal.edu.co

**Keywords:** anthocyanins, antioxidant activity, bioactive compounds, colorants, fruit juices, polyphenols, wild blueberry

## Abstract

Andean blueberry (*Vaccinium meridionale* Swartz) fruits are an underutilized source of anthocyanins and other valuable bioactive phytochemicals. The purpose of this work was to obtain Andean blueberry juice powders via freeze-drying processing and evaluate the effect of maltodextrin as a drying aid on their physicochemical, technological, microstructural, and bioactive characteristics. Andean blueberry juices were mixed with variable proportions of maltodextrin (20–50%); freeze-dried; and characterized in terms of their tristimulus color, Fourier transform infrared spectra (FTIR), moisture content, water activity, morphology, water solubility, flow properties, total polyphenols and anthocyanins content, and DPPH^•^-scavenging capacity. The powders obtained presented suitable characteristics in terms of their water activity (<0.5), solubility (>90%), and bioactive compound recovery (>70% for total phenolics, and >60% for total monomeric anthocyanins), with antioxidant activities up to 4 mg equivalent of gallic acid/g of dry matter. Although an increased content of maltodextrin resulted in lower concentrations of phytochemicals, as expected, it also favored an increased % recovery (over 90% of total phenolics at the highest maltodextrin proportion) and improved their flow properties. Freeze-dried juice powders are a potential alternative for the stabilization and value addition of this fruit as a new source of functionality for processed foods.

## 1. Introduction

The use of natural ingredients has received widespread attention in recent years due to its high demand in different industrial fields such as food, pharmaceutics, and cosmetics [1]. In particular, in the food industry there is a growing interest in the extraction, characterization, and stabilization of new natural ingredients that can be further incorporated into functional foods.

The genus *Vaccinium* (family Ericaceae) comprises about 450 species, known for their high content of phytochemicals [2]. Andean blueberry (*Vaccinium meridionale* Swartz) is a wild shrub with few commercial exploitations that grows in the Andean region of South America at 2300–3300 m above sea level (m.a.s.l.) [3]. Several studies have reported that Andean blueberry fruits are a rich source of bioactive compounds, such as anthocyanins, flavonoids, and phenolic acids, which have been associated with antioxidant, anticarcinogenic, and anti-inflammatory properties [4,5,6].

Andean blueberry fruits are commonly marketed either fresh or processed into jellies and jams, with a very low supply of derived value-added products [4]. Andean blueberry juice is very attractive to small-scale food processors of rural areas because it is easy to produce and has adequate sensory properties [7]. Indeed, several studies had reported that Andean blueberry juice has a high potential for use as an antioxidant additive or functional ingredient in foods because it is rich in polyphenols and has a high antioxidant capacity [3,4]. However, as with most fruit juices Andean blueberry juices are susceptible to microbial spoilage and degradation due to their high moisture content. Moreover, the anthocyanin content and antioxidant capacity of Andean blueberry juice are significantly reduced during storage [3,8]. Therefore, it is necessary to develop strategies to increase their storage life.

The dehydration of fruit juices is a promising approach to obtain highly stable dried powders that are more resistant to microbial and oxidative degradation, light in weight, and readily soluble; furthermore, it enables room temperature storage over longer periods [9]. Several drying techniques are available for the production of food powders at an industrial scale, with freeze drying and spray-drying being the most successful methods for fruit juice powder production [10].

Freeze drying is a low-temperature dehydration process that removes ice or frozen solvent through sublimation and has the advantages of being flexible, straightforward, and easily scalable [11,12]. Additionally, freeze-drying is suitable for the processing of heat-sensitive active compounds, because substances are not exposed to high temperatures, unlike in conventional air-drying and spray-drying [12,13]. Nonetheless, fruit juices are generally difficult to dry due to their high content of sugars and organic acids, which make the dried products extremely hygroscopic, adhesive, and very susceptible to degradation during storage. Therefore, food-grade drying aids (carriers), such as maltodextrin and gum Arabic, have been added to spray-dried or freeze-dried fruit juices [9,14].

Maltodextrins are polysaccharides obtained by the partial enzymatic or acid hydrolysis of starch that consist of β-D-glucose units linked mainly by glycosidic bonds α(1→4). These low-cost carbohydrate polymers feature good film-forming properties, a high water solubility, and a neutral taste and aroma [10]. It has been reported that maltodextrins allow obtaining juice powders with a good stability against oxidation, ease of handling, improved solubility, and extended shelf life [10]. Several studies have been carried out to evaluate the optimal drying conditions for different fruit juices. Lachowicz et al. [15] studied the effect of maltodextrin and inulin on the protection of natural antioxidants in powders made of Saskatoon berry fruit, juice, and pomace, finding that the freeze-drying process using these wall materials led to the highest content of polyphenolic compounds and antioxidant activity. Pudziuvelyte et al. [16] encapsulated *Elsholtzia ciliata ethanolic* extract by freeze-drying using skim milk, sodium caseinate, gum Arabic, maltodextrin, β-cyclodextrin, and resistant maltodextrin, alone or in mixtures of two or four encapsulants. The highest value of encapsulation efficiency of phenolic compounds was obtained for powders prepared using sodium caseinate alone or in a mixture with resistant maltodextrin and maltodextrin.

The aim of the current work was to develop freeze-dried Andean blueberry juice powders for potential applications as ingredients with bioactive characteristics. The effect of the addition of different maltodextrin concentrations (20–50%) on the moisture content, water activity, water solubility, bulk density, flow properties, color attributes, morphology, polyphenols and anthocyanins content, and antioxidant capacity of the juice powders was evaluated. To the best of our knowledge, this is the first report on powders from freeze-dried Andean blueberry juice.

## 2. Results and Discussion

### 2.1. Andean Blueberry Juice Properties

The physicochemical properties of the Andean blueberry juice used in this work are presented in Table 1. The juice was characterized by a relatively high content of soluble solids (13.27 °Brix) and high concentrations of antioxidants, both total phenolic compounds and anthocyanins, which are expected to be more stable at the mildly acidic pH of this product (2.91) [17]. In fact, the juice presented a DPPH radical scavenging capacity of 19.1 mg GAE/g dw. These characteristics, along with its deep ruby color, similar to that of Tannat or Merlot wines, and sweet characteristic taste highlight that this product is suitable for consumption as fresh juice with antioxidant features or for addition as a functional ingredient. The composition of berry fruits and juices depends on the cultivar, maturity stage, and agro-climatic conditions. In particular, the characteristics of Andean blueberry could be more variable because it grows as a wild shrub. According to previous reports, Andean blueberry fruits at maturity stages have a soluble solids content ranging between 12 and 18 °Brix, a pH between 2.5 and 3.0, a dry solid content between 17% and 23%, total monomeric anthocyanins between ~329 ± 28 mg cyd-3-glu/100 g, and phenolic compounds ~758.6 ± 62.3 mg gallic acid equivalent/100 g [3,18]. Franco et al. found similar values to those reported in this study (Table 1) when they evaluated the physicochemical properties of Andean blueberry nectar [19]. Casati et al. [20] evaluated the physicochemical characteristics from berries juices cultivated in Argentina (blueberry, elderberry, blackcurrant, and maqui berry), finding soluble solids contents ranging between 9.0 and 14.8 °Brix, a pH between 3.4 and 4.2, a water activity between 0.983 and 0.989, total polyphenol contents between 2970 and 9340, and total monomeric anthocyanins contents between 288 and 1795.4 mg cyd-3-glu/L.

### 2.2. Physicochemical and Morphological Properties of the Juice Freeze-Dried Powders

**Figure 1:**
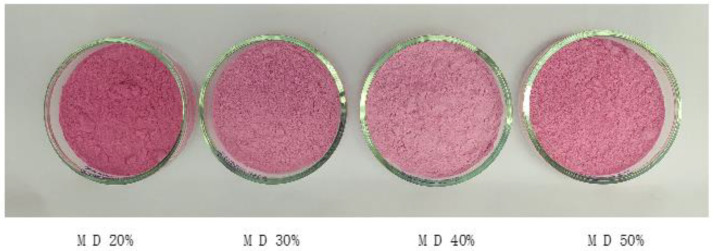
Images of freeze-dried powders obtained using different maltodextrin (MD) concentrations.

Mixtures of the extracted blueberry juices and maltodextrin with no further ingredients were prepared and subjected to freeze drying processing to obtain juice powders. The general appearance of the juice powders obtained with the addition of maltodextrin is shown in Figure 1; unlike the maltodextrin-free freeze-dried juice (Figure S1, Supplementary Material), these powders were solid, easily manageable, and did not adhere to solid surfaces.

The CIELAB color coordinates of Andean blueberry juice powders are given in Table 2. Compared to the deep ruby color of the pure juice, the colors of the powders were dark fuchsia pink. The lightness of the Andean blueberry juice powders increased when the maltodextrin concentration increased from 20% to 40% (i.e., a lighter color). Above this material amount, a slight decrease in this parameter was observed (Table 2). Other authors have also described a higher lightness after the addition of higher maltodextrin amounts [21].

Hue angle (*h*) and chroma are a very important color attributes that characterize the perception and the purity and intensity of the color, respectively. All the powders showed low *h* values (i.e., closest to the angle for red (0°)), as expected in this type of products (Table 2).

The samples with maltodextrin at 20% showed the higher values of chroma, indicating the highest intensity of color. Above this concentration, non-significant differences between the chroma values of the samples were observed (Table 2). As expected, the samples with a higher Andean blueberry juice concentration (maltodextrin at 20%) showed the higher values of coordinate a* (indicating redness) (Table 2).

The moisture content and the water activity of the Andean blueberry freeze-dried powders ranged from 4.0% to 9.0% and from 0.2 to 0.5, respectively (Table 3). The longer shelf life of dried products is closely related to lower moisture content and water activity. It has been reported that, in dried food powders with a low water activity (aw < 0.6), no microbial proliferation occurs, and the product could be considered fully stable in that respect [22]. Similar results were obtained for sumac extract powders by Caliskan et al. [14].

**Figure 2:**
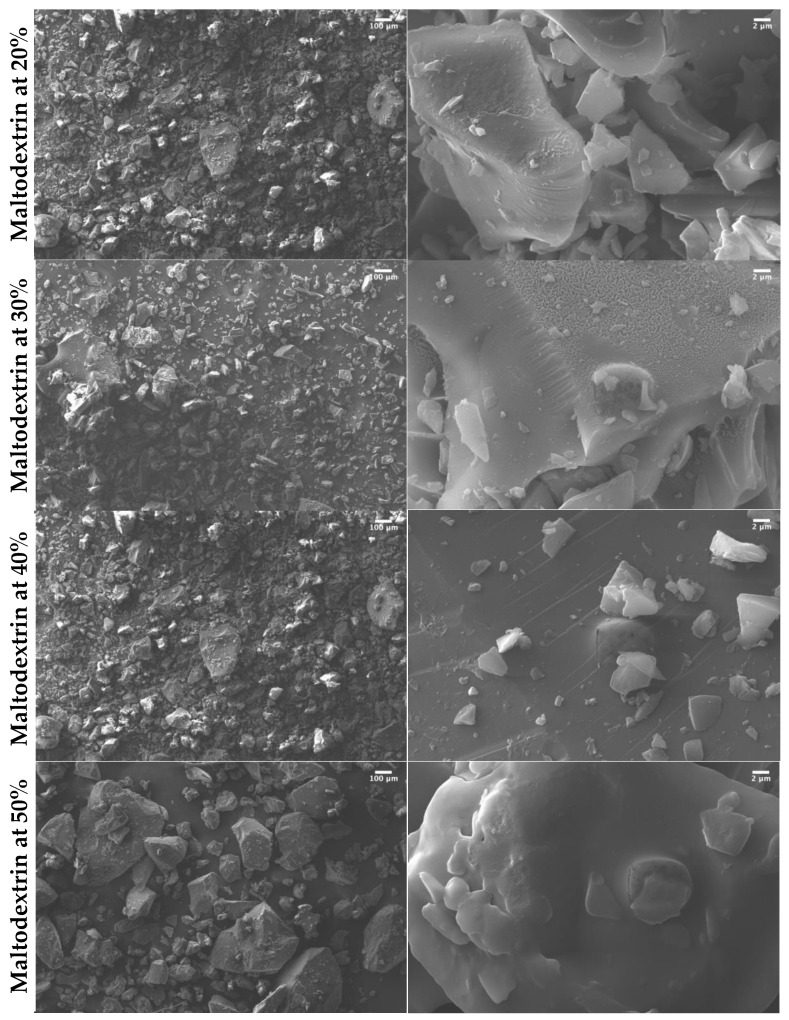
Scanning electron microscopy (SEM) images of freeze-dried powders obtained using different wall materials and mixtures.

The microstructure of the encapsulated dried fruit extracts is relevant to their water reconstitution behavior, flowability, and other techno-functional characteristics; although it is mainly dependent on the type of encapsulant and the drying technique, it can vary according to the extract-encapsulant interactions and ratio [23]. Figure 2 shows SEM images of the freeze-dried Andean blueberry juice powders obtained using different concentrations of maltodextrin. All the images show the typical morphology of freeze-dried powders, with an irregular glassy shape [12,16]. In this case, the microstructure of the powders appeared to be mainly defined by the characteristic crystallinity of the maltodextrin, even at lower (20%) encapsulant concentrations. This indicates that the thermodynamic compatibility between maltodextrin and the Andean blueberry juice solids allows for obtaining amorphous but microscopically homogeneous encapsulated materials via freeze-drying, and suggests that the macroscopical and techno-functional properties of these powders will be defined by those of the encapsulant, thus enhancing their manageability as a powdery food ingredient. The observed differences in the particle sizes might be attributed to the grounding of the freezing-dried cakes [24]. González-Ortega et al. [25] encapsulated olive leaf extract by freeze-drying, reporting that a porous cake was obtained due to the sublimation of the ice, giving rise to a structure made of a glassy matrix containing air cells whose size and shape depended on the processing conditions used and the composition of the initial system.

**Figure 3:**
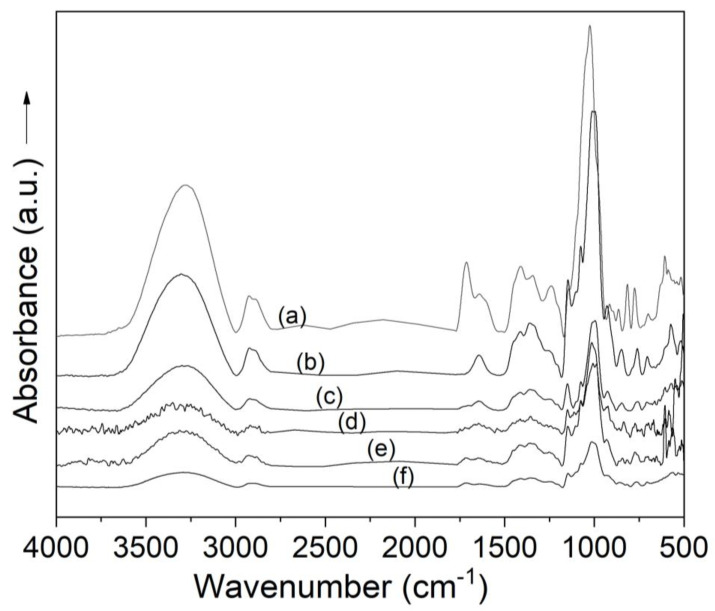
FTIR spectra of Andean blueberry juice (**a**); maltodextrin (**b**); and Andean blueberry juice powders with maltodextrin at 20% (**c**), 30% (**d**), 40% (**e**), and 50% (**f**).

To evaluate the effect of freeze drying on the spectral characteristic of the juices and possible interactions between the Andean blueberry juice solids and maltodextrin at the processing conditions, the absorbance spectra of the powders in the mid-infrared region of electromagnetic radiation were recorded. Figure 3 shows the infrared spectra (4000–500 cm^−1^) of the Andean blueberry juice powders obtained with different concentrations of maltodextrin. The spectra of the Andean blueberry juice and the maltodextrin are shown for comparison. Andean blueberry juices featured characteristic bands at 1712 cm^−1^ corresponding to –C=O bonds, and at 1630 cm^−1^ and 1521 cm^−1^ previously associated with the C=C vibrations of polyphenolic compounds from anthocyanin-rich berry extracts [26]. The IR spectra of all Andean blueberry juice powders showed the characteristic bands of maltodextrin at 3300 cm^−1^ (O–H stretching), 2905 cm^−1^ (C–H_2_ asymmetric stretching), 1641 cm^−1^ (free carboxyl groups), 1150 cm^−1^ (C–O stretching), 1005 cm^−1^ (C–O stretching), and 929 cm^−1^ (C–O–C stretching of glycosidic bonds; CH_2_ out of plane bending) [27]. The characteristic absorption bands of Andean blueberry juice were also detected in the juice in the freeze-dried powders (Figure 3).

The absorbance of the bands located at 1630, 1521, 1410^,^ and 1024 cm^−1^, associated with phenolic compounds and in particular with the presence of anthocyanins [26], were quantitatively correlated with the juice content of the powders, which indicates that FTIR-ATR measurements could be used as fast technique to assess the actual juice content in this type of ingredients. The absence of bands unrelated to either maltodextrin or Andean blueberry juice suggest that maltodextrin was an inert wall material with no observable chemical interactions with the Andean blueberry juices.

### 2.3. Technological Features of the Juice Freeze-Dried Powders

Table 4 shows the flow properties of Andean blueberry juice freeze-dried powders obtained with different concentrations of maltodextrin. The bulk density of the freeze-dried powders increased with the increase in the maltodextrin concentration—i.e., the samples with lower maltodextrin concentrations showed higher cohesiveness. The results obtained were similar to those determined for freeze-dried powders containing cinnamon infusions (536–554 kg × m^−3^) [28] and sea buckthorn juice (512.7 kg × m^−3^) [21]. All the powders showed an increase in their bulk density due to the tapping suggesting the presence of attractive forces and friction [29]. On the other hand, the samples with a maltodextrin concentration at 40% and 50% showed a slight decrease in the Hausner ratio and a lower Carr index and angle of repose than the powders with 20% and 30% of wall material, indicating better flow properties (Table 4). In general, higher maltodextrin proportions improved the flowability of the freeze-dried juices. Similar observations were reported by Caliskan et al. [14] when analyzing the effect of different amounts of maltodextrin addition on the powder properties of freeze-dried sumac extract powders.

The water solubility of the freeze-dried powders is significant for its incorporation in food systems [14]. All the freeze-dried Andean blueberry juice powders showed a similar water solubility—i.e., close to 93%—regardless of the maltodextrin concentration added (Table 3). This behavior can be attributed to the high solubility of maltodextrin in water and is in accordance with the microstructure similarity of the powders obtained at different encapsulant proportions in the SEM observations. Franceschinis et al. [30] reported a water solubility (%) of almost 100% for powders from blackberry juice obtained by freeze and spray-drying. Several authors have reported that the water solubility of freeze-dried powders depends on the morphology, the particle size, the inter-particle voids of powders, and the properties of juice and carrier agents [29].

### 2.4. Bioactive Characteristics of Andean Blueberry Powders with Different Maltodextrin Additions

**Figure 4:**
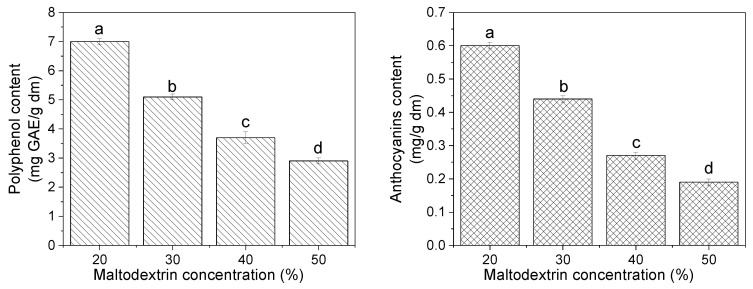
Total polyphenol content and total monomeric anthocyanin of freeze-dried Andean blueberry juice powders with different concentrations of maltodextrin. Values with different superscript letters are significantly different (*p* < 0.05).

The determination of the active compound content of freeze-dried fruit juice powders is important for estimating the powder amount necessary to reach a determined active compounds level in a food formulation. Figure 4 shows the total polyphenol content and total monomeric anthocyanin of freeze-dried Andean blueberry juice powders with different concentrations of maltodextrin. In general, a decrease in the total polyphenol content and the total monomeric anthocyanin of the powders was observed as the maltodextrin concentration in the formulations increased—i.e., as the juice amount in the powders decreased. The content of total phenolic compound ranged between 2.9 and 7.0 mg GAE/g of dry matter, while the monomeric anthocyanins content ranged between 0.19 and 0.60 mg cyd-3-glu/g of dry matter. The results obtained in this study were close to the ones previously reported by Casati et al. [20] for freeze-dried blueberry (*Vaccinium corymbosum*) powders: phenolic contents of 7.69 mg GAE/g and total monomeric anthocyanins content of 0.74 mg/g.

**Figure 5:**
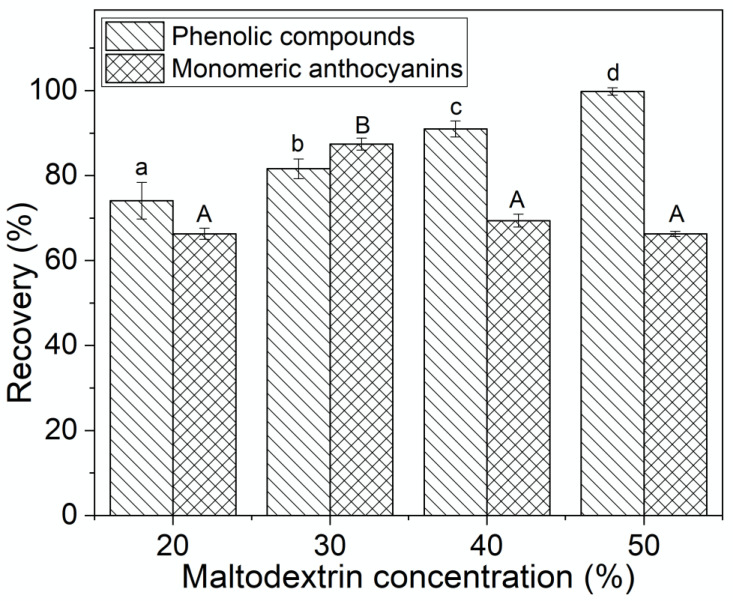
Recovery (%) of the total polyphenol content and total monomeric anthocyanin in freeze-dried Andean blueberry juice powders with different concentrations of maltodextrin. Values with different superscript letters are significantly different (*p* < 0.05).

On the other hand, the retention of phenolic compounds of the freeze-dried powders was significantly improved with increasing the maltodextrin concentration in the formulations (Figure 5). In all cases, retention percentages of phenolic compounds greater than 70% were obtained, with a higher recovery in the powders with 50% of maltodextrin. The percentage of recovery of monomeric anthocyanins increased significantly when the maltodextrin concentration increased from 20% to 30% (Figure 5). However, above this concentration a significant decrease in anthocyanin retention was observed. Romero-González et al. [31], when working with maqui juice freeze-dried powders, reported that the anthocyanin efficiency values decreased at a higher proportion of added polysaccharides (maltodextrin, gum Arabic, inulin, and their blends). Fraceschinis et al. [30], when working with freeze-dried blackberry powders, reported percentages of the retention of polyphenols and anthocyanins of 73% and 75%, respectively.

**Figure 6:**
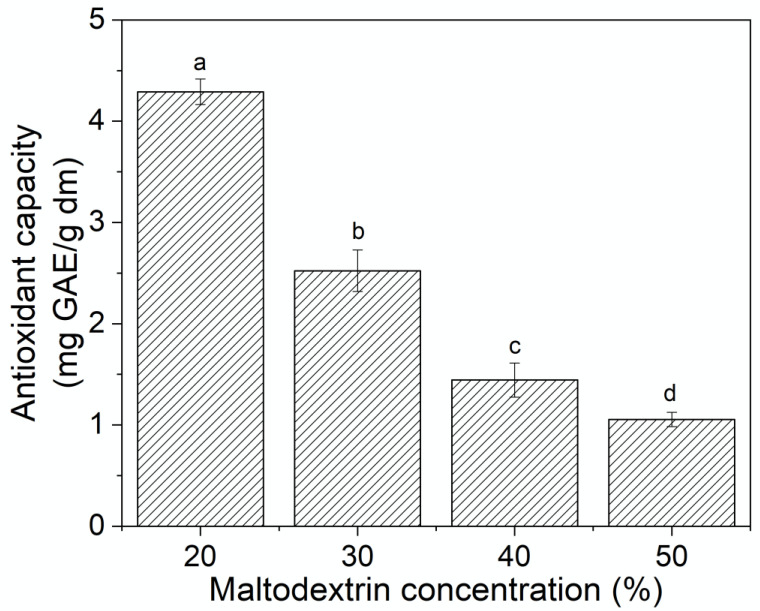
Antioxidant activity (DPPH^•^ scavenging capacity) of the freeze-dried powders obtained using different maltodextrin (MD) concentrations. Values with different superscript letters are significantly different (*p* < 0.05).

Figure 6 shows the antioxidant activity of the freeze-dried powders obtained using different maltodextrin (MD) concentrations. The DPPH^•^-scavenging activity of the freeze-dried powders increased as the maltodextrin concentration in the formulation decreased. In this sense, a high correlation (R^2^ = 0,99) between the polyphenol content and the antioxidant activity of the powders was obtained, indicating a strong influence of the phenolic compound on this parameter. This correlation between polyphenol content and antioxidant capacity was also observed by Garrido-Makinistian et al. [32] in maqui powders obtained by spray-drying.

Garzón et al. reported that the high antioxidant activity of Andean blueberry fruits could be due to the high concentration and the chemical structure of its phenolic compounds. They detected in this fruit through high-performance liquid chromatography with photodiode array detection (HPLC-DAD) and HPLC-electrospray ionization tandem mass spectrometry (ESI-MS/MS) the presence of bioactive compounds with a strong antioxidant activity, such as monoglucosides of cyanidin and delphinidin, chlorogenic acid, and quercetin [4].

## 3. Materials and Methods

### 3.1. Materials

Andean blueberries (*Vaccinium meridionale* Swart) maturity stage 5 (100% dark purple) were obtained in Ráquira (Boyacá, Colombia) at 2150 m.a.s.l. The berries were examined previous to its use to separate fruits with physical, mechanical, or microbial damage. The fruits were washed and disinfected with a 100 mg L^−1^ of chlorine solution.

Maltodextrin (MD) dextrose equivalent (DE) 18–22 from Tecnas S.A. (Medellín, Colombia) was used as a carrier. Folin–Ciocalteu reagent was purchased from Panreac (Barcelona, Spain) and gallic acid was purchased from Merck (Darmstadt, Germany). 2,2-diphenyl-1-picrylhydrazyl (DPPH^•^) reagent was purchased from Sigma-Aldrich (St. Louis, MO, USA). All the chemicals used were of analytical grade.

### 3.2. Preparation of Andean Bluebery Juice and Freeze-Drying Formulations

Andean blueberry juices were squeezed from crushed fruits using a juice press extractor and vacuum filtered through Whatman paper N °1.

Four formulations for freeze drying were obtained blending the juice (J) with maltodextrin (MD) in the following ratios (J:MD): 80:20 (MD20), 70:30 (MD30), 60:40 (MD40), and 50:50 (MD50). Maltodextrin was dissolved in the Andean blueberry juices under constant stirring using a EUROSTAR 20 vertical agitator at 800 rpm (IKA, Staufen, Germany). The homogenized formulations were poured on aluminum trays for the freezing drying process.

In preliminary experiments, the freeze drying of pure juices (100:0) and its blend with maltodextrin at 10% (90:10) was also assayed, but these formulations did not lead to stable juice powders (Table S1, Supplementary Material).

### 3.3. Freeze Drying

All the formulations were frozen at −20 °C for 24 h and then dried using a BUCHI Lyovapor L-200 freeze dryer (Flawil, Switzerland). It was operated at −55 °C at a chamber pressure of 0.1 mbar for 48 h. The freeze-drying cakes were grounded to obtain powders and stored in hermetic flask until use.

### 3.4. pH and Soluble Solids Content

The pH was assessed using a digital pH meter (Oakton Instruments, Vernon Hills, IL, USA) (AOAC 981.12). The soluble solids content was measured in the fruit juice using an Atago refractometer model PR 101 (Atago CO., Tokyo, Japan) and expressed as °Brix (AOAC 932.12).

### 3.5. Fourier Transform Infrared Spectroscopy (FTIR)

FTIR analysis was performed using FT/IR-4100 equipment (JASCO, Hachioji, Tokyo, Japan) equipped with a diamond single reflection attenuated total reflectance (ATR) module. Portions of the samples (approximately 10 mg) were placed on the ATR accessory and analyzed under reflectance mode. A total of 24 spectra per sample were acquired with 24 scans per spectrum with a spectral resolution of 4 cm^−1^ in the spectral interval 4000–450 cm^−1^. The measured spectra were recorded and pre-treated with the built-in procedures for water elimination using the software Spectra Manager (v.2.7, JASCO Hachioji, Tokyo, Japan).

### 3.6. Color

Color was measured using a tristimulus Minolta colorimeter (Konica-Minolta CR-10, Osaka, Japan) and was reported in CIELab parameters (L*, a* and b* values), where L* was used to denote lightness, a* redness (+) and greenness (−), and b* yellowness (+) and blueness (−).

Hue angle (*h*) and chroma values were calculated using the following equations:

(1) $$h=\tan^{-1}\left(\frac{b^{*}}{a^{*}}\right),$$

(2) $$\;\mathrm{Croma}={\left[a^{*2}+b^{*2}\right]}^{1/2}\;$$

### 3.7. Morphological Analysis

The morphological characteristics of the freeze-dried powders were investigated by Scanning Electron Microscopy (SEM) using a ZEISS EVO MA10 microscope (Carl Zeiss SMT Ltd., Cambridge, UK). The powders were attached to stubs, coated with a layer of gold, and examined using an acceleration voltage of 20 kV.

### 3.8. Moisture Content and Water Activity

Humidity content (%) was measured gravimetrically, drying the freeze-dried powders in an oven at 105 °C until they reached a constant weight (AOAC, 1998). Values of water activity (a_w_) were determined using an AquaLab Serie 3 TE (Pullman, WA, USA) apparatus (AOAC, 1998).

### 3.9. Water-Solubility

Water solubility was determined by blending 1 g of the freeze-dried powders with 100 mL of distilled water at room temperature with continuous stirring at 1000 rpm for 5 min (IKA RT5 magnetic stirring, Staufen, Germany). The rehydrated juice was centrifuged at 1500 rpm for 5 min and the supernatant was dried at 105 °C until constant weight. The dry weight was used to calculate solubility as a percentage.

### 3.10. Total Polyhenols Content

The total polyphenols content of Andean blueberry juice and powders was determined by the Folin–Ciocalteu method [33]. Briefly, 400 μL of reconstituted juice was mixed with 2 mL of Folin–Ciocalteu reagent (1:10 diluted). Then, 1.6 mL of sodium carbonate (7% *w*/*v*) was added to each sample. After 30 min, the absorbance was measured at 760 nm using a spectrophotometer (X-ma 1200 Human Corporation, Loughborough, UK). The results were expressed as gallic acid equivalents (GAE) per gram of dry solids.

### 3.11. Total Monomeric Anthocyanins Content

The total monomeric anthocyanins content of Andean blueberry juice and powders was measured by the pH differential method [34]. Absorbencies were read at 520 and 700 nm. The anthocyanin concentration was calculated and expressed as cyanidin 3-glucoside (cyd-3-glu) using an extinction coefficient (ε) of 26,900 L cm^−1^ × mol^−1^ and a molecular weight of 449.2 g/mol.

### 3.12. Active Compounds Recovery (%)

The recovery (%) of phenolic compounds and monomeric anthocyanins after the freeze drying process was calculated with the following equation [35]:

(3) $$\mathrm{Recovery}\;\left(\%\right)=\frac{L_{c}}{L_{0}}\times 100.$$

where L_c_ and L_0_ are the total phenolic or monomeric anthocyanin content of the freeze-dried powders and the infeed dispersion, respectively.

### 3.13. DPPH^•^-Scavenging Activity

Antioxidant activity was tested as described in Brand-Williams et al. [36]. A volume of 100 μL of each reconstituted juice was mixed with 3.9 mL of 1,1-diphenyl- 2-picrylhydrazyl (DPPH^•^) ethanol solution (25 mg DPPH^•^/L). Absorbance was determined at 515 nm until the reaction reached a plateau. A calibration curve was performed using gallic acid as a standard. The results were expressed as the mg GAE per gram of dry solids.

### 3.14. Flow Properties

The loose bulk density was determined by pouring a known mass of freeze-dried powders delivered freely by gravity into a measuring cylinder and calculated by dividing the mass by the bulk volume. The tapped bulk density was calculated from the weight of powder and the volume occupied in the cylinder after being hand tapped until a constant value was reached [29,37].

The Hausner ratio and the compressibility index were estimated according to the procedures presented in López Córdoba et al. [37]

The angle of repose was determined by pouring a known mass of freeze-dried powder through a funnel located at a fixed height on a graph paper flat horizontal surface and measuring the height (h) and radius (r) of the conical pile formed. The tangent of the angle of repose is given by the h/r ratio [38].

### 3.15. Statistical Analysis

The statistical analysis was performed using the Minitab v.16 statistical software (State College, PA, USA). Analysis of variance (ANOVA) and Tukey’s pairwise comparisons were carried out using a level of 95% confidence. The experiments were performed at least in triplicate, and the data were reported as means ± standard deviations.

## 4. Conclusions

Maltodextrin was found a suitable wall material for the stabilization of Andean blueberry juice via freeze-drying because it allowed obtaining powders with good handling properties and desirable features, such as low water activity and a high water solubility. Besides this, maltodextrin is an inert wall material that does not have chemical interactions with Andean blueberry juices.

The amount of maltodextrin used in the formulations significantly affected the lightness, the bulk density, the polyphenol and monomeric anthocyanins content, and the antioxidant activity of the freeze-dried Andean blueberry juice powders. The products with maltodextrin at 30% and 50% showed higher monomeric anthocyanin and polyphenol recoveries, respectively. The antioxidant activity of the freeze-dried Andean blueberry juice was highly correlated with its content of phenolic compounds. The produced powders could be potentially employed as functional ingredients for the formulation of new value-added foods.

## Figures and Tables

**Table 1:** Physicochemical properties of the Andean blueberry juice.

Physicochemical Property	Value
Soluble solids content (°Brix)	13.27 ± 0.05
Dry solid content (%)	11.6 ± 0.3
pH	2.91 ± 0.07
Water activity	0.97 ± 0.01
Color coordinates (CIELAB)	L* = 22.7 ± 0.4
	a* = 22.5 ± 0.4
	b* = 7.8 ± 0.5
	*h* = 19.2 ± 1.9
	c = 23.2 ± 0.8
Total polyphenol content (mg GAE/L)	2032.5 ± 41.7
Monomeric anthocyanin content (mg cyd-3-glu/L)	371.5 ± 20.1
Antioxidant capacity (mg GAE/ g dw)	19.1 ± 0.3

mg GAE: milligrams equivalents of gallic acid; g dw: grams of dry matter; mg cyd-3-glu: milligrams of cyanidin 3-glucoside.

**Table 2:** Color parameter of Andean blueberry juice freeze-dried powders obtained with different concentrations of maltodextrin.

Maltodextrin Concentration (%)	L*	a*	b*	*h*	Chroma
20	47.2 ± 0.8 ^a^	42.3 ± 0.5 ^a^	2.6 ± 0.4 ^a^	3.6 ± 0.5 ^a^	42.3 ± 0.5 ^a^
30	54.2 ± 0.6 ^b^	36.6 ± 0.3 ^b^	1.4 ± 0.1 ^b^	2.2 ± 0.2 ^b^	36.6 ± 0.3 ^b^
40	56.7 ± 1.1 ^c^	35.6 ± 0.6 ^b^	3.4 ± 0.4 ^a^	5.5 ± 0.6 ^c^	35.8 ± 0.6 ^b^
50	52.7 ± 1.7 ^b^	38.0 ± 2.7 ^b^	2.9 ± 0.4 ^a^	4.3 ± 0.7 ^b^	38.1 ± 2.7 ^b^

Different letters within the same column indicate significant differences (*p* < 0.05).

**Table 3:** Moisture content, water activity, and water solubility of Andean blueberry juice freeze-dried powders obtained with different concentrations of maltodextrin.

Maltodextrin Concentration (%)	Moisture Content (%)	Water Activity (a_w_)	Water Solubility (%)
20	6.1 ± 0.4 ^a^	0.31 ± 0.03 ^a^	94.6 ± 0.4 ^a^
30	4.3 ± 0.1 ^b^	0.27 ± 0.01 ^a^	93.2 ± 0.9 ^a^
40	5.4 ± 0.1 ^a,b^	0.41 ± 0.05 ^b^	92.8 ± 0.8 ^a^
50	8.6 ± 0.3 ^c^	0.52 ± 0.01 ^c^	91.1 ± 0.5 ^a^

Different letters within the same column indicate significant differences (*p* < 0.05).

**Table 4:** Flow properties of Andean blueberry juice freeze-dried powders obtained with different concentrations of maltodextrin.

Maltodextrin Concentration (%)	Bulk Density kg × m^−3^	Tapped Density kg × m^−3^	Hausner Ratio	Carr Index (%)	Angle of Repose (°)
20	470 ± 24 ^a^	545 ± 44 ^a^	1.2 ± 0.1 ^a,b^	20.7 ± 2.6 ^a^	35.4 ± 0.3 ^a^
30	502 ± 20 ^a^	615 ± 17 ^b^	1.2 ± 0.1 ^a^	15.6 ± 0.4 ^b^	37.0 ± 0.5 ^a^
40	585 ± 35 ^b^	674 ± 39 ^c^	1.1 ± 0.1 ^a,b^	11.6 ± 0.7 ^c^	36.3 ± 1.1 ^a^
50	595 ± 41 ^b^	650 ± 43 ^b,c^	1.1 ± 0.1 ^b^	6.1 ± 0.2 ^d^	27.0 ± 0.9 ^b^

Different letters within the same column indicate significant differences (*p* < 0.05).

## Supplementary Materials

The following are available online, Figure S1: Images of the maltodextrin-free freeze dried-powder, Table S1: Physicochemical properties of the maltodextrin-free freeze dried-powder.

## References

[1] Da Silva B.V., Barreira J.C., Oliveira M.B.P. (2016). Natural phytochemicals and probiotics as bioactive ingredients for functional foods: Extraction, biochemistry and protected-delivery technologies. Trends Food Sci. Technol..

[2] Song G.-Q., Hancock J.F., Kole C. (2011). Vaccinium. Wild Crop Relatives: Genomic and Breeding Resources.

[3] Celis M.E.M., Franco Tobón Y.N., Agudelo C., Arango S.S., Rojano B., Yahia E.M. (2017). Andean Berry (Vaccinium meridionale Swartz). Fruit and Vegetable Phytochemicals: Chemestry and Human Health.

[4] Garzón G.A., Narváez C.E., Riedl K.M., Schwartz S.J. (2010). Chemical composition, anthocyanins, non-anthocyanin phenolics and antioxidant activity of wild bilberry (Vaccinium meridionale Swartz) from Colombia. Food Chem..

[5] González M., Samudio I., Sequeda-Castañeda L.G., Celis C., Iglesias J., Morales L. (2017). Cytotoxic and antioxidant capacity of extracts from Vaccinium meridionale Swartz (Ericaceae) in transformed leukemic cell lines. J. Appl. Pharm. Sci..

[6] Maldonado-Celis M.E., Arango-Varela S.S., Rojano B.A. (2014). Free radical scavenging capacity and cytotoxic and antiproliferative effects of *Vaccinium meridionale* Sw. agains colon cancer cell lines. Rev. Cuba. Plantas Med..

[7] Agudelo C.D., Ceballos N., Gómez-García A., Maldonado-Celis M.E. (2018). Andean Berry (Vaccinium meridionale Swartz) Juice improves plasma antioxidant capacity and IL-6 levels in healthy people with dietary risk factors for colorectal cancer. J. Berry Res..

[8] Celli G.B., Dibazar R., Ghanem A., Brooks M.S.-L. (2016). Degradation kinetics of anthocyanins in freeze-dried microencapsulates from lowbush blueberries (Vaccinium angustifolium Aiton) and prediction of shelf-life. Dry. Technol..

[9] Shishir M.R.I., Chen W. (2017). Trends of spray drying: A critical review on drying of fruit and vegetable juices. Trends Food Sci. Technol..

[10] Nicoletti Telis V.R., Martínez-Navarrete N., Nicoletti Telis V.R. (2012). Biopolymers Used as Drying Aids in Spray-Drying and Freeze-Drying of Fruit Juices and Pulps. Biopolymer Engineering in Food Processing.

[11] Delshadi R., Bahrami A., Tafti A.G., Barba F.J., Williams L.L. (2020). Micro and nano-encapsulation of vegetable and essential oils to develop functional food products with improved nutritional profiles. Trends Food Sci. Technol..

[12] Fredes C., Becerra C., Parada J., Robert P. (2018). The Microencapsulation of Maqui (*Aristotelia chilensis* (Mol.) Stuntz) Juice by Spray-Drying and Freeze-Drying Produces Powders with Similar Anthocyanin Stability and Bioaccessibility. Molecules.

[13] Aprodu I., Milea Ș.A., Anghel R.-M., Enachi E., Barbu V., Crăciunescu O., Râpeanu G., Bahrim G.E., Oancea A., Stănciuc N. (2019). New Functional Ingredients Based on Microencapsulation of Aqueous Anthocyanin-Rich Extracts Derived from Black Rice (*Oryza sativa* L.). Molecules.

[14] Caliskan G., Dirim S.N. (2016). The effect of different drying processes and the amounts of maltodextrin addition on the powder properties of sumac extract powders. Powder Technol..

[15] Lachowicz S., Michalska-Ciechanowska A., Oszmiański J. (2020). The Impact of Maltodextrin and Inulin on the Protection of Natural Antioxidants in Powders Made of Saskatoon Berry Fruit, Juice, and Pomace as Functional Food Ingredients. Molecules.

[16] Pudziuvelyte L., Marksa M., Sosnowska K., Winnicka K., Morkuniene R., Bernatoniene J. (2020). Freeze-Drying Technique for Microencapsulation of Elsholtzia ciliata Ethanolic Extract Using Different Coating Materials. Molecules.

[17] Fossen T., Cabrita L., Andersen O.M. (1998). Colour and stability of pure anthocyanins influenced by pH including the alkaline region. Food Chem..

[18] Garzón G.A., Soto C.Y., López-R M., Riedl K.M., Browmiller C.R., Howard L. (2020). Phenolic profile, in vitro antimicrobial activity and antioxidant capacity of Vaccinium meridionale Swartz pomace. Heliyon.

[19] Franco Tobon Y.N., Rojano B.A., Arbeláez Alzate A.F., Saavedra Morales D.M., Celis Maldonado M.E. (2016). Efecto del tiempo de almacenamiento sobre las características fisicoquímicas, antioxidantes y antiproliferativa de néctar de agraz (Vaccinium meridionale Swartz). Arch. Latinoam. Nutr..

[20] Casati C.B., Baeza R., Sánchez V. (2019). Physicochemical properties and bioactive compounds content in encapsulated freeze-dried powders obtained from blueberry, elderberry, blackcurrant and maqui berry. J. Berry Res..

[21] Tkacz K., Wojdyło A., Michalska-Ciechanowska A., Turkiewicz I.P., Lech K., Nowicka P. (2020). Influence Carrier Agents, Drying Methods, Storage Time on Physico-Chemical Properties and Bioactive Potential of Encapsulated Sea Buckthorn Juice Powders. Molecules.

[22] Tapia M.S., Alzamora S.M., Chirife J. (2007). Effects of Water Activity (aw) on Microbial Stability: As a Hurdle in Food Preservation. Water Act. Foods.

[23] Sarabandi K., Peighambardoust S.H., Sadeghi Mahoonak A.R., Samaei S.P. (2018). Effect of different carriers on microstructure and physical characteristics of spray dried apple juice concentrate. J. Food Sci. Technol..

[24] Khazaei K.M., Jafari S.M., Ghorbani M., Hemmati Kakhki A. (2014). Application of maltodextrin and gum Arabic in microencapsulation of saffron petal’s anthocyanins and evaluating their storage stability and color. Carbohydr. Polym..

[25] González-Ortega R., Faieta M., Di Mattia C.D., Valbonetti L., Pittia P. (2020). Microencapsulation of olive leaf extract by freeze-drying: Effect of carrier composition on process efficiency and technological properties of the powders. J. Food Eng..

[26] Alzate-Arbeláez A.F., Dorta E., López-Alarcón C., Cortés F.B., Rojano B.A. (2019). Immobilization of Andean berry (Vaccinium meridionale) polyphenols on nanocellulose isolated from banana residues: A natural food additive with antioxidant properties. Food Chem..

[27] Ballesteros L.F., Ramirez M.J., Orrego C.E., Teixeira J.A., Mussatto S.I. (2017). Encapsulation of antioxidant phenolic compounds extracted from spent coffee grounds by freeze-drying and spray-drying using different coating materials. Food Chem..

[28] Santiago-Adame R., Medina-Torres L., Gallegos-Infante J.A., Calderas F., González-Laredo R.F., Rocha-Guzmán N.E., Ochoa-Martínez L.A., Bernad-Bernad M.J. (2015). Spray drying-microencapsulation of cinnamon infusions (Cinnamomum zeylanicum) with maltodextrin. LWT Food Sci. Technol..

[29] López-Córdoba A., Goyanes S. (2017). Food Powder Properties. Ref. Modul. Food Sci..

[30] Franceschinis L., Salvatori D.M., Sosa N., Schebor C. (2014). Physical and Functional Properties of Blackberry Freeze- and Spray-Dried Powders. Dry. Technol..

[31] Romero-González J., Shun Ah-Hen K., Lemus-Mondaca R., Muñoz-Fariña O. (2020). Total phenolics, anthocyanin profile and antioxidant activity of maqui, *Aristotelia chilensis* (Mol.) Stuntz, berries extract in freeze-dried polysaccharides microcapsules. Food Chem..

[32] Garrido Makinistian F., Sette P., Gallo L., Bucalá V., Salvatori D. (2019). Optimized aqueous extracts of maqui (*Aristotelia chilensis*) suitable for powder production. J. Food Sci. Technol..

[33] Singleton V.L., Orthofer R., Lamuela-Raventos R.M., Lester Packer L. (1999). Analysis of total phenols and other oxidation substrates and antioxidants by means of Folin-Ciocalteu reagent. Methods in Enzymology (Oxidants and Antioxidants, Part A).

[34] Lee J., Durst R.W., Wrolstad R.E. (2005). Determination of Total Monomeric Anthocyanin Pigment Content of Fruit Juices, Beverages, Natural Colorants, and Wines by the pH Differential Method: Collaborative Study. J. AOAC Int..

[35] Fang Z., Bhandari B. (2011). Effect of spray drying and storage on the stability of bayberry polyphenols. Food Chem..

[36] Brand-Williams W., Cuvelier M.E., Berset C. (1995). Use of a free radical method to evaluate antioxidant activity. LWT Food Sci. Technol..

[37] López-Córdoba A., Deladino L., Agudelo-Mesa L., Martino M. (2014). Yerba mate antioxidant powders obtained by co-crystallization: Stability during storage. J. Food Eng..

[38] Rattes A.L.R., Oliveira W.P. (2007). Spray drying conditions and encapsulating composition effects on formation and properties of sodium diclofenac microparticles. Powder Technol..

