# Supplementary material for: New Freeze-Dried Andean Blueberry Juice Powders for Potential Application as Functional Food Ingredients: Effect of Maltodextrin on Bioactive and Morphological Features
Source: Molecules. 2020 Nov 30;25(23):5635. doi: 10.3390/molecules25235635 (PMC7730302; doi:10.3390/molecules25235635)
Supplement: Supplementary file 1 [file molecules-25-05635-s001.pdf]

molecules

MDPI

Supplementary material

New Freeze-Dried Andean Blueberry Juice Powders For Potential Application As Functional Food Ingredients: Effect of Maltodextrin on Bioactive and Morphological Features

Mauren Estupiñan-Amaya [1], Carlos Alberto Fuenmayor [2] and Alex López-Córdoba [1,*]

1 Facultad Seccional Duitama, Escuela de Administración de Empresas Agropecuarias, Universidad Pedagógica y Tecnológica de Colombia, Carrera 18 con Calle 22 Duitama 150461, Boyacá, Colombia
2 Instituto de Ciencia y Tecnología de Alimentos (ICTA), Universidad Nacional de Colombia, Av. Carrera 30 # 45-03, 111321 Bogotá, Colombia; cafuenmayorb@unal.edu.co

* Correspondence: alex.lopez01@uptc.edu.co (A.L.-C.); Tel.: +57-8-7604100

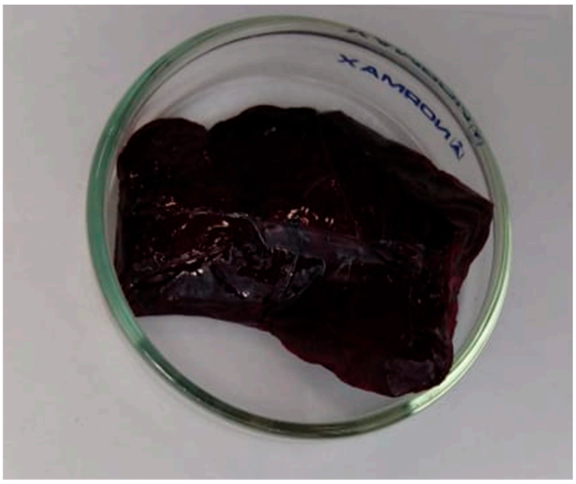

Figure S1. Images of the maltodextrin-free freeze dried-powder

Table S1 Physicochemical properties of the maltodextrin-free freeze dried-powder

Physicochemical property	Value
Water activity	0.35±0.01
Moisture content (%)	11.2±0.3
Flowability index	non-free flowing powders
Water solubility (%)	87.2±0.5
Color coordinates (CIELAB)	L*= 22.5±0.5<br>a*= 11.3±0.5<br>b*= 7.0±0.8
